# Soluble ST2 as a Biomarker for Predicting Right Ventricular Dysfunction in Acute Pulmonary Embolism

**DOI:** 10.3390/jcm13237211

**Published:** 2024-11-27

**Authors:** Muhammet Uyanik, Ahmet Cinar, Omer Gedikli, Tibel Tuna, Bahattin Avci

**Affiliations:** 1 Department of Cardiology, Carsamba State Hospital, 55500 Samsun, Turkey; 2 Department of Cardiology, Merzifon State Hospital, 05300 Amasya, Turkey; ahmetcinaar@gmail.com; 3 Department of Cardiology, Faculty of Medicine, Ondokuz Mayis University, 55270 Samsun, Turkey; drgedikli@hotmail.com; 4 Department of Pulmonary Diseases, Faculty of Medicine, Ondokuz Mayis University, 55270 Samsun, Turkey; tibeltuna@hotmail.com; 5 Department of Biochemistry, Faculty of Medicine, Ondokuz Mayis University, 55270 Samsun, Turkey; bahattinavci@hotmail.com

**Keywords:** sST2, pulmonary embolism, pulmonary hypertension, PESI

## Abstract

**Introduction:** Suppression of Tumorigenicity 2 (ST2), a member of the interleukin-1 (IL-1) superfamily, is recognized as an important biomarker in inflammatory responses and cardiovascular diseases. Elevated serum levels of sST2 have prognostic value, particularly in cases of cardiac stress such as heart failure and acute pulmonary embolism (APE). We aimed to assess ST2 levels as a potential biomarker for right heart dysfunction in APE patients, particularly in the context of its limited predictive value for mortality and risk stratification. **Methods:** Patients diagnosed with APE confirmed via computed tomography pulmonary angiography (CTPA) were enrolled in this study. To ensure the specificity of sST2 elevation to APE, patients with other conditions known to cause elevated sST2 levels were excluded. **Results:** After pre-clinical evaluation, 66 patients diagnosed with APE who met the study criteria, and 62 healthy subjects in the control group, were included in this study. sST2 levels were positively correlated with APE. **Conclusions:** In patients diagnosed with APE, sST2 levels had high sensitivity. sST2 levels are elevated in APE and are associated with right ventricular dysfunction, but do not independently predict mortality or risk stratification based on Pulmonary Embolism Severity Index (PESI) scores.

## 1. Introduction

Acute pulmonary embolism (APE) is the third most common acute cardiovascular disease worldwide, with high mortality rates [1,2]. Therefore, timely and accurate assessment of APE severity is crucial for effective patient management. Stratification of patients typically relies on a combination of hemodynamic parameters, evidence of right ventricular (RV) overload through echocardiography, computed tomography pulmonary angiography (CTPA), arterial blood gas analysis, and various biomarkers indicative of RV ischemia and injury. PESI score is frequently recommended in clinical guidelines as a tool for risk stratification, aiding in early mortality risk assessment [3]. Traditionally, biomarkers such as troponin, which indicates myocardial damage, and B-type natriuretic peptide (BNP), associated with right ventricular dysfunction, have been extensively studied for their roles in risk stratification. However, there remains a need for additional biomarkers that can offer enhanced predictive value and further refine risk stratification models.

Interleukin-33 (IL-33) is a member of the IL-1 family that promotes innate and adaptive inflammatory responses [4]. It is released in response to cell death and damage [5]. Serum tumorigenicity 2 protein (ST2) is a member of the IL-1 receptor-like 1 (IL-1RL1) family. ST2 exists in three isoforms: ST2L, ST2V, and the soluble form circulating in blood [6]. ST2L is a ligand that mediates the IL-33 proinflammatory effect and is present in the cell membrane [5]. Serum sST2 is an isomer with a smaller molecular structure that is released outside the cell [7]. sST2 limits inflammatory responses by blocking IL-33 binding to ST2L, playing a key role in the fibrosis process [7]. In cardiac diseases, elevated sST2 is linked to fibrosis and adverse clinical outcomes, a pattern also observed in pulmonary conditions.

During early inflammation, proinflammatory cytokines like IL-1a, IL-1b, and IL-6 stimulate sST2 secretion [8,9]. Most sST2 is released from inflammatory cells, epithelial cells of the respiratory system, alveoli, fibroblasts, and myocytes [5]. sST2 has various and complex roles in the pathogenesis of different cancers [5]. It also plays a role in allergic asthma, chronic obstructive pulmonary disease (COPD), idiopathic pulmonary fibrosis, and other immune-affecting diseases [10]. sST2 is secreted in response to mechanical tension in myocytes and fibroblasts [11]. It is a known predictor of cardiovascular morbidities and mortalities, especially heart failure [12]. Elevated sST2 levels suggest increased fibrosis and are linked to adverse clinical outcomes [13]. As in cardiac diseases, increased sST2 levels are associated with negative outcomes in pulmonary diseases [14].

sST2 has emerged as an important biomarker associated with inflammatory responses and thrombotic processes. A study by Memon et al. highlights sST2 as a potential diagnostic and prognostic tool in conditions such as deep vein thrombosis [15]. Similarly, research by Cabrera-Garcia et al. in COVID-19 patients underscores the role of sST2 as an indicator of hypercoagulability and inflammation, demonstrating its potential in clinical monitoring and thrombotic risk assessment [16]. These findings support the utility of sST2 in monitoring and risk stratification for conditions like acute pulmonary embolism (APE) and related disorders.

Considering these mechanisms, this study aims to investigate the relationship between sST2 levels, which can be measured from blood serum, and morbidity, mortality, and PESI risk scores in patients diagnosed with APE. Understanding the role of sST2 in APE could contribute significantly to new management and risk stratification strategies. This study addresses a gap by assessing sST2 as a potential biomarker for right heart dysfunction in APE patients, particularly regarding its limited predictive value for mortality and risk stratification.

## 2. Patients and Methods

Patients admitted to the emergency department of our tertiary hospital with suspected APE, whose diagnoses were confirmed between November 2020 and January 2022, were enrolled in this study. The diagnosis of APE was confirmed using pulmonary CT angiography. Control group participants were randomly selected from volunteers who matched the demographic (age and sex) and clinical characteristics of the APE patients, with no conditions known to increase ST2 levels. A total of 66 APE patients and 62 controls were included after applying exclusion criteria.

Demographic, clinical, and laboratory data, as well as radiological images, were recorded for each patient. Risk factors such as hypertension, diabetes mellitus, hypercholesterolemia, smoking status, and a history of lung disease were documented. Subgroup analysis of APE patients was conducted based on the 2019 ESC/ERS (European Society of Cardiology/European Respiratory Society) guidelines. Patients were classified as low-, intermediate-, or high-risk according to the PESI score [3]. Given that sST2 levels can be influenced by cardiovascular diseases, a specialist cardiologist evaluated the ECG, echocardiography, and cardiac history of all participants. Patients with decompensated heart failure, acute coronary syndrome within the last 3 months, cancer, active infectious diseases, rheumatoid or inflammatory diseases, asthma, or COPD were excluded from this study. This study complied with the Declaration of Helsinki, and the Ondokuz Mayis University Clinical Research Ethics Committee approved the study protocol (OMU KAEK 2020/670). Written informed consent was obtained from all participants.

Blood samples were collected from both patients and controls for complete blood count (CBC), sedimentation, C-reactive protein (CRP), creatinine, aspartate transaminase (AST), alanine transaminase (ALT), lactate dehydrogenase (LDH), D-dimer, and sST2 levels at admission.

CBC was determined using a Sysmex automated hematology analyzer XN-100 (Sysmex, Kobe, Japan). Serum D-dimer levels were determined by Afias-6 Boditech (Boditech Med Inc, Chuncheon, Republic of Korea), and the erythrocyte sedimentation rate (ESR) was determined using a Sistat ESR 120 (Sistat Diagnosis and Treatment Systems Inc, Ankara, Turkey) device. Serum AST, ALT, LDH, creatinine, and CRP concentrations were measured with a Roche Hitachi Cobas 8000 (Hitachi High-Tech Corporation, Tokyo, Japan) device using Roche Diagnostics GmbH kits (Roche GmbH, Mannheim, Germany). All assays were performed in accordance with the manufacturer’s instructions.

For sST2, blood samples freshly collected in EDTA-coated tubes were centrifuged at 3000 rpm (Shimadzu UV160A, S. No: 28006648, Shimadzu Corporation, Kyoto, Japan) for 20 min, and the serum was stored at −80 °C for further analysis. On the day of evaluation, the serum was melted at room temperature.

The concentrations of human soluble ST2 (sST2) in serum were measured using commercially available enzyme-linked immunosorbent assay (ELISA) kits (SunredBio, catalog no: 201-12-1724, Shanghai, China). sST2 levels were expressed as ng/mL. The mean inter-assay coefficient of variation (CV) % and intra-assay CV % were <5.26% and <6.1%, respectively. All assays were performed in accordance with the manufacturer’s instructions.

Chest CT was performed using a commercial multidetector CT scanner (Canon, Aquillion Prime SP, Canon Medical Systems, Canon, Ōtawara, Japan), and lung involvement was evaluated by two experienced radiologists.

### Statistical Analysis

Statistical analyses were conducted using SPSS Statistics for Windows, version 21.0. Data were presented as mean ± standard deviation (SD), median (min–max), or frequency (%). The Shapiro–Wilk test was used to assess the normal distribution of quantitative variables. Data from the two groups were analyzed using Student’s t test for normally distributed data and the Mann–Whitney test for non-normally distributed data. One-way ANOVA, followed by Tukey and Tamhane tests for post hoc comparisons, was used to analyze normally distributed data among three groups. Kruskal–Wallis’s variance analysis was used for non-normally distributed data among three groups, followed by Bonferroni-corrected Mann–Whitney U tests for post hoc comparisons. Frequencies were compared using Pearson’s chi-square test. The area under the ROC curve (AUC) was evaluated as a measure of diagnostic test discrimination, with confidence intervals calculated for AUC. Sensitivity and specificity values were assessed, with statistical significance set at *p* < 0.05.

## 3. Results

The study cohort consisted of 66 patients with confirmed APE, comprising 41 females and 25 males, with a mean age of 67.9 years (range 24–80). Baseline characteristics, clinical symptoms, comorbidities, and echocardiographic parameters at admission are detailed in Table 1. Predisposing factors were identified in nine patients: four were immobile postoperatively, two were mobile postoperatively, and three were immobile due to various sequelae. No metabolic or hematological predisposing factors were identified. Deep venous thrombosis (DVT) was detected in 12 (18%) patients via lower-extremity Doppler ultrasonography at admission. Patients were stratified into risk categories based on the current ESC/ERS guidelines: 13 (19.7%) were high-risk, 17 (25.8%) intermediate-risk, and 36 (54.5%) low-risk. Treatment was administered according to these guidelines, with five patients receiving thrombolysis (alteplase 0.6 mg/kg, max 50 mg, via 15 min IV infusion). During the in-hospital or 30-day observation period, six patients (6.1%) died, all of whom were presenting with right ventricular (RV) heart failure. A composite endpoint, including hemodynamic collapse, cardiopulmonary resuscitation, need for intravenous catecholamine infusion, intubation, or major bleeding, was observed in five patients (7.5%).

The laboratory findings of APE patients revealed significant differences compared to the control group, particularly with elevated levels of creatinine, ALT, hemoglobin, and CRP. Higher WBC and neutrophil percentages observed in the APE group indicate a more pronounced inflammatory response in these patients. Blood gas analysis showed that APE patients had lower pO₂ and higher FiO₂ levels than the control group, which is suggestive of respiratory impairment associated with pulmonary embolism. Table 2 demonstrates the significant differences in laboratory findings between APE patients and controls, emphasizing the inflammatory and hemodynamic burden in APE.

**Figure 1:**
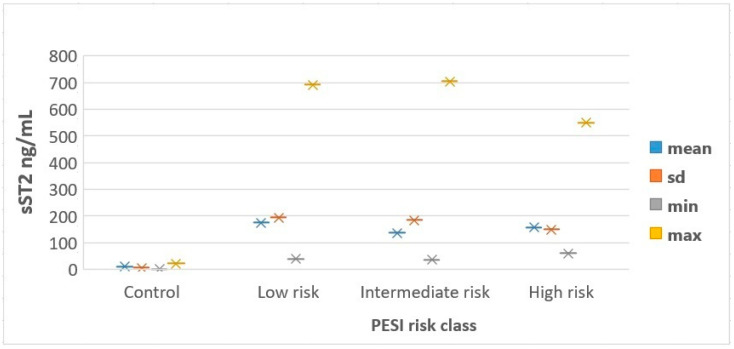
Distribution of sST2 levels according to PESI risk classes. PESI—Pulmonary Embolism Severity Index; sd—standard deviation; sST2—soluble Suppression of Tumorigenicity 2.

sST2 concentration was higher in APE patients than in the control group (162.1 ± 182.4 (37.1–704.7) and 10.6 ± 6.6 ng/mL, respectively; *p*< 0.001). sST2 is not reflected in the severity of APE: 175.8 ± 194.8 (39.6–691.6) ng/mL in low-risk APE, 136.7 ± 184.6 (37.1–704.7) ng/mL in intermediate-risk APE, and 157.4 ± 150.0 (61.1–550.1) ng/mL in high-risk APE, Figure 1. Although sST2 levels were higher in APE patients compared to controls, the lack of a clear trend among PESI risk groups suggests that sST2 may not reliably indicate the severity of APE. The sST2 blood concentration was related to the biochemical and echocardiographic indices of right ventricular overload. In patients with right ventricular dysfunction detected on echocardiography, plasma sST2 concentrations were higher than in subjects without RV dysfunction (152.1 [94.1–704.7], *p* = 0.046). There was a significant negative correlation between sST2 levels and TAPSE (r = −0.35, *p* = 0.03). A positive correlation was found between sST2 levels and RV diameter (r = 0.40, *p* = 0.02). A strong positive correlation was observed between sST2 and PASB (r = 0.45, *p* = 0.01). No significant correlations were found between sST2 levels and EF (r = −0.15, *p* = 0.25). Higher sST2 levels were associated with RV dysfunction, suggesting its role in monitoring right heart strain in APE patients. sST2 levels correlated with troponin-I levels (r = 0.248, *p* = 0.044). This suggests a modest correlation between sST2 levels and markers of myocardial injury, indicating potential clinical relevance. There was no correlation between sST2 plasma levels and D-dimer levels in our study (r = −0.214, *p* = 0.085). The median sST2 levels in patients who died within 30 days after APE diagnosis were not higher than those in survivors, at 130.6 ng/mL (55.1–250.5) and 132.4 ng/mL (37.1–704.7), respectively (*p* = 0.41). Similarly, patients who experienced an adverse outcome did not have significantly higher sST2 plasma levels than those with a favorable course, with levels of 133.2 ng/mL (55.1–210.7) and 137 ng/mL (37.1–704.7), respectively (*p* = 0.71). However, despite the lack of significant association with mortality or adverse outcomes, higher sST2 levels were correlated with longer hospitalization duration (r = 0.447, *p* < 0.001).

**Figure 2:**
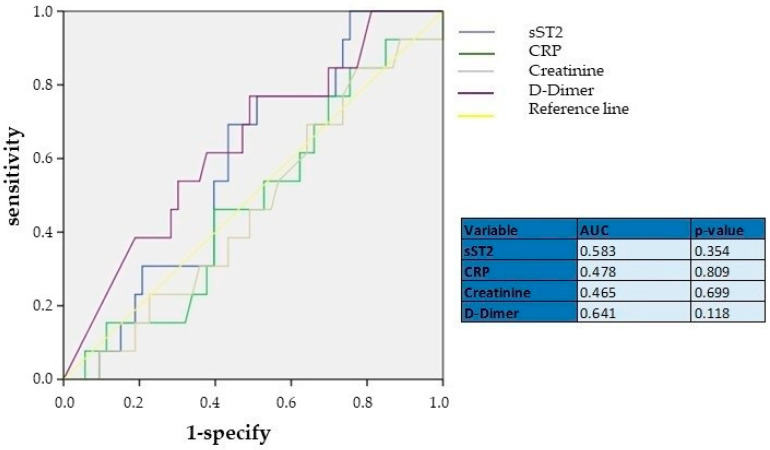
ROC curve analyses showing the diagnostic performance of sST2, CRP, creatinine, and D-dimer. AUC—area under curve; CRP—C-reactive protein; sST2—Suppression of Tumorigenicity.

We performed another ROC curve analysis to determine the diagnostic role of sST2 in predicting severe PESI scores (>106) (Figure 2). As shown, sST2 was not a parameter with a significant discriminative capacity for worse PE prognosis, with an AUC of 0.583 (*p* = 0.354), indicating its limited use as a prognostic biomarker. Similarly, CRP and creatinine also showed low discriminative power, with AUCs of 0.478 (*p* = 0.809) and 0.465 (*p* = 0.699), respectively, suggesting that these biomarkers may not be reliable indicators for assessing APE prognosis when used independently. In contrast, D-dimer, which had an AUC of 0.641 (*p* = 0.114), showed relatively better performance compared to sST2, CRP, and creatinine, though still below the threshold for strong prognostic tools (AUC > 0.7). This suggests that D-dimer remains a relatively useful diagnostic marker, while sST2 and CRP do not demonstrate significant discriminative power for APE prognosis.

## 4. Discussion

This study aimed to elucidate the relationship between serum sST2 levels, PESI risk scores, and mortality rates in patients with APE. Our findings demonstrated a significant elevation of sST2 levels in APE patients compared to healthy controls. However, we observed no significant differences in sST2 levels across PESI low-, intermediate-, and high-risk groups, nor between survivors and non-survivors. We found a relationship between deterioration of right ventricular function indicators and sST2. This aligns, indicating that, while sST2 reflects RV strain, it may not independently predict APE severity or clinical outcomes.

The primary pathophysiological role of sST2 in cardiac disease is related to its protective interaction in the IL-33/sST2 axis. This interaction protects cardiac cells against stress by limiting apoptosis and modulating inflammatory responses. However, elevated sST2 levels antagonize the cardioprotective effect of IL-33, weakening this axis and contributing to the progression of myocardial damage [11]. This biomarker regulates inflammatory responses in cardiomyocytes under mechanical stress through interaction with IL-33, thereby indicating cardiac stress.

sST2 has been studied in many acute and chronic cardiovascular diseases. Among patients with suspected aortic dissection in the emergency department, sST2 showed superior overall diagnostic performance for D-dimer or cardiac troponin I [17]. ST2 is associated with pulmonary complications and mortality in polytraumatized patients [18]. Shimpo et al. investigated sST2 levels in 810 patients in the Thrombolysis In Myocardial Infarction (TIMI) 14 and Enoxaparin and TNK-tPA With or Without GPIIb/IIIa Inhibitor as Reperfusion Strategy in STEMI (ENTIRE)-TIMI 23 clinical trials and found that elevated ST2 levels at admission were associated with death or new-onset heart failure at 1 month [19]. Kohli et al. showed that among patients in the MERLIN TIMI 36 trial, sST2 concentrations were associated with an increased risk of heart failure or adverse cardiac events at 30 days and 1 year [20].

The role of sST2 in various pathophysiological processes and its clinical significance in heart failure have been the focus of numerous studies in recent years. Beyond traditional biomarkers (such as BNP and NT-proBNP), sST2 presents a unique alternative for prognosis. For instance, a study by Gaggin et al. demonstrated that sST2 is effective in predicting mortality in both acute and chronic heart failure, making it a valuable indicator for assessing both short- and long-term outcomes [21]. sST2 levels have been observed to rise parallel to progression or worsening clinical status in heart failure patients, suggesting their use as a dynamic biomarker for disease management [22]. Comparative analyses of emerging biomarkers, such as galectin-3 and sST2, have shown that sST2 offers superior prognostic value in patients with heart failure [23]. In addition to diagnosis and risk stratification, the use of sST2 in clinical settings holds considerable potential for monitoring treatment response. A study by McGinn et al. suggests that sST2 may even be used for clinical monitoring in pediatric heart failure [24]. These findings indicate that sST2 may be a trackable biomarker for chronic and acute conditions, aligning with the disease progression rate and clinical outcomes. Previous studies have demonstrated a correlation between sST2 levels and right ventricular failure arising from various etiologies [25]. According to our literature review, this study is the first to directly demonstrate that sST2 serves as an indicator of right ventricular dysfunction in APE.

Petramala et al. demonstrated that sST2 levels were significantly elevated in APE patients and associated with more severe clinical findings, such as higher PESI scores, serum lactate, and CRP levels [26]. Notably, sST2 levels above 35 ng/mL were linked to worse prognosis and had superior predictive power compared to other biomarkers. Similarly, Gunes et al. reported that elevated sST2 levels were significantly associated with increased 6-month mortality and repeated cardiovascular hospitalizations in APE patients [27].

However, our study indicates that while sST2 reflects right ventricular dysfunction in a more controlled cohort, it does not independently predict in-hospital clinical outcomes. This discrepancy may stem from differences in the characteristics of the study populations. Our study was conducted on a more homogeneous group by excluding comorbidities such as cancer, COPD, and advanced heart failure, potentially contributing to the lower correlation observed between sST2 and clinical severity. The more pronounced prognostic value of sST2 in APE patients with multiple comorbidities may be attributed to its role in inflammatory and thrombotic processes. These findings highlight that the prognostic value of sST2 can vary depending on patient population and study methodology. This underscores the need for further investigation of sST2 in diverse patient profiles and risk categories. Future studies should focus on developing more comprehensive risk stratification and management strategies by combining sST2 with other biomarkers and imaging techniques. Large studies are needed to evaluate the correlation between sST2 and PESI. In this context, assessing sST2 alongside the PECSS score, potentially due to its association with RV, could prove more beneficial for evaluating acute hemodynamic impacts and guiding thrombolytic treatment decisions.

The relationship between sST2 and CTEPH, which develops in the context of chronic pulmonary embolism, has been investigated. Kerkütlüoğlu et al. found that high sST2 levels increased the likelihood of developing Chronic Thromboembolic Pulmonary Hypertension (CTEPH) [28]. Interleukin 33 (IL-33) acts by suppressing the tumorigenicity 2 receptor (ST2) and is highly expressed in the barrier regions. It disrupts the balance between widespread inflammation and tissue generation and causes remodeling, which is a distinctive property of fibrosis [11]. Counter-regulatory mechanisms are initiated to limit the proinflammatory actions of IL-33, as indicated by an increase in the decoy receptor for IL-33, the soluble form of ST2 [29]. These roles of sST2 in inflammation and fibrosis may be associated with worse clinical outcomes in patients with CTEPH. In a comparative study on biomarkers, elevated levels of GDF-15 and sST2 were observed in patients with CTEPH, correlating with significant hemodynamic impairment [30]. Following balloon pulmonary angioplasty in inoperable CTEPH patients, both sST2 and GDF-15 levels demonstrated a marked reduction. This decrease in serum biomarker levels was accompanied by hemodynamic improvements and enhanced functional capacity. These findings indicate that sST2 may serve as a promising biomarker for monitoring treatment response in pulmonary embolism and related clinical conditions. In our study, sST2 was evaluated in the hospital. As its long-term fibrotic effects have not yet emerged, there may be no significant difference between the risk groups. This highlights sST2′s potential role as a predictor of long-term clinical outcomes. In our literature review, we did not find any studies evaluating the treatment response in acute pulmonary embolism (APE). Future studies should focus on assessing treatment response in APE.

sST2 has recently emerged as a notable biomarker in cardiology and pulmonary diseases. Extensive studies have primarily focused on its association with heart failure, with substantial evidence supporting its role as a prognostic biomarker and the exploration of its integration into multi-marker strategies. Our findings are consistent with prior research demonstrating the link between sST2 and ventricular dysfunction in cardiovascular conditions. The primary cause of mortality in APE is right ventricular (RV) failure due to pressure overload. Current studies on pulmonary embolism predominantly assess short-term mortality risk and disease severity. However, the role of sST2 as a pre-existing risk factor in the general population, particularly in the context of postoperative APE development, remains unexplored. Comprehensive studies involving large patient cohorts to evaluate sST2 as a diagnostic marker for ruling out APE have not been conducted. Similarly, combining the evaluation of sST2 with the YEARS algorithm may provide new insights to facilitate diagnostic assessments. Clinical utility as a marker for assessing treatment response in APE has yet to be investigated. These findings underscore the potential of sST2 as a valuable biomarker in the management of APE and highlight the need for further research to validate its clinical utility.

### Study Limitation

This study had several limitations. First, the exact onset of APE symptoms was difficult to determine in many patients, which may have affected the timing of biomarker measurement. Second, our exclusion criteria, particularly the exclusion of patients with chronic comorbidities like cancer or COPD, may have limited the generalizability of our findings to a broader APE population. Lastly, the relatively small sample size may have reduced the statistical power to detect significant differences in sST2 levels across different risk categories.

## 5. Conclusions

In conclusion, while sST2 is significantly elevated in APE and correlates with right ventricular dysfunction, it does not independently predict mortality or clinical outcomes based on PESI scores. sST2′s role as a complementary biomarker, particularly in combination with other markers, should be further evaluated to improve risk stratification in PE patients. Future studies should focus on integrating sST2 into a multimodal approach, combining biochemical markers with imaging techniques, to enhance early identification and management of PE patients.

## Figures and Tables

**Table 1:** Baseline characteristics.

	PE (*n* = 66)	Control (*n* = 62)	*p*-Value
Age (year)	67.9 ± 13.7 (24–93)	52.8 ± 15.7 (22–90)	<0.001 ^a,^*
Gender			
Male	25 (37.9)	35 (56.5)	0.035 ^c,^*
Female	41 (62.1)	27 (43.5)	
BMI (kg/m^2^)	28.6 ± 3.6 (20.8–37.2)	28.9 ± 3.8 (21.0–37.0)	0.610 ^b^
Chronic disease			
Hypertension	41 (62.1)	15 (24.2)	<0.001 ^c,^*
Allergic asthma	11 (16.7)	5 (8.1)	0.141 ^c^
Diabetes mellitus	11 (16.7)	4 (6.5)	0.073 ^c^
Hyperlipidemia	5 (7.6)	5 (8.1)	1.000 ^d^
Smoking	14 (21.2)	20 (32.3)	0.157 ^c^
Beta blocker	17 (25.8)	10 (16.1)	0.182 ^c^
CCB	15 (22.7)	3 (4.8)	0.004 ^c,^*
ACE-I	6 (9.1)	10 (16.1)	0.229 ^c^
ARB	22 (33.3)	4 (6.5)	<0.001 ^c,^*
Antithrombotic	11 (16.7)	2 (3.2)	0.012 ^c,^*
EF, %	56.6 ± 6.0 (25–70)	57.4 ± 4.4 (45–70)	0.854 ^a^
PASB by TRPG	38.3 ± 17.6 (15–120)	18.0 ± 4.4 (10–30)	<0.001 ^a,^*
RV diameter	30.1 ± 5.5 (21–46)	24.0 ± 3.7 (16–30)	<0.001 ^a,^*
TAPSE	17 ± 4.1	21.9 ± 3.13	<0.0002
RV overload	42 (63.6%)		
Heart rate	100 (85–122)	75 (52–109)	<0.001
Respiratory rate	20	14	<0.001
Systolic blood pressure	138	130	0.47
Diastolic blood pressure	84	80	0.51
PESI	88.7 ± 35.4 (24–223)		

* Continuous variables are presented as “mean ± standard deviation (minimum–maximum)” and categoric variables are presented as “number (column percentage)”. ^a^ Mann–Whitney U test; ^b^ Student’s *t* test; ^c^ Pearson chi-square test; ^d^ Fisher’s Definitive Test. ACE-I—Angiotensin converting enzyme inhibitor; ARB—Angiotensin receptor blockers; CCB—Calcium channel blocker; EF—ejection fraction; PASB—Pulmonary artery systolic pressure; PESI—Pulmonary Embolism Severity Index; RV—right ventricle; LV—left ventricle; TRPG—tricuspid valve pressure gradient. (RV overload was diagnosed when echocardiography showed the following: RV free wall hypokinesis, RV/LV > 1.0, and AcT (acceleration time) < 60 ms of pulmonary ejection according to 2019 ESC Acute PE guidelines).

**Table 2:** Laboratory findings.

	APE	Control	*p*
Creatinine	1.09 ± 0.52 (0.39–3.61)	0.95 ± 0.48 (0.58–4.00)	0.022 ^a,^*
ALT	25.3 ± 27.5 (4.4–140.0)	27.2 ± 19.5 (2.5–120.0)	0.011 ^a,^*
AST	27.3 ± 20.2 (5.0–107.0)	24.8 ± 10.7 (11.2–73.0)	0.288 ^a^
Hemoglobin	12.0 ± 2.0 (7.3–17.1)	13.7 ± 2.0 (8.1–17.3)	<0.001 ^b,^*
WBC	10.6 ± 3.6 (4.0–19.5)	7.2 ± 2.7 (4.0–17.0)	<0.001 ^a,^*
Neutrophil (mm^3^)	8.0 ± 3.4 (2.9–18.5)	4.3 ± 2.2 (1.7–13.1)	<0.001 ^a,^*
Neutrophil, %	73.3 ± 10.0 (42.3–94.9)	58.0 ± 13.3 (31–90)	<0.001 ^b,^*
Lymphocytes (mm^3^)	1.72 ± 0.85 (0.30–4.04)	2.23 ± 1.14 (0.50–7.94)	0.006 ^a,^*
Lymphocytes, %	17.2 ± 7.6 (1.8–47.2)	31.0 ± 11.3 (5.3–60.9)	<0.001 ^b,^*
Thrombocyte	260.6 ± 128.3 (101–801)	248.9 ± 81.3 (120–552)	0.574 ^a,^*
CRP	66.8 ± 57.3 (0.6–185.0)	3.31 ± 2.38 (0.6–14.9)	<0.001 ^a,^*
PaO_2_ (mmHg)	80.2 ± 6.09 (74–95)	96.2 *±* 8.1 (94–105)	<0.001 ^b,^*
PaCO_2_ (mmHg)	35.5 ± 3.9 (28–42)	38.3 *±* 3.9 (34–45)	0.002 ^b,^*
FiO_2_ (%)	35 ± 4.8 (28–45)	21 *±* 2.60	<0.001 ^b,^*
SpO_2_ (%)	92 ± 3.7 (85–97)	97.1 *±* 1.5 (95–99)	<0.001 ^b,^*
P/F ratio	230 ± 38 (150–350)	452 *±* 40 (400–500)	<0.001 ^b,^*
Lactate (mmol/L)	2.2 ± 0.6 (1.5–4.0)	1.0 *±* 0.3 (0.7–1.5)	<0.001 ^b,^*
D-Dimer	4977.6 ± 3826.2 (123–10,000)	594.1 ± 887.4 (92.9–4126.0)	<0.001 ^a,^*
Troponin-I (ng/mL)	241.9 ± 491.5 (0.1–2613.0)	10.1 ± 7.4	<0.001 ^a,^*
sST2	162.1 ± 182.4 (37.1–704.7)	10.1 ± 6.6 (1.0–21.6)	<0.001 ^a,^*

* Variables are presented as “mean ± standard deviation (minimum–maximum)”. ^a^ Mann–Whitney U Test; ^b^ Student’s *t* test. ALT—Alanine transaminase; APE—acute pulmonary embolism; AST—Aspartate transaminase; CRP—C-reactive protein; sST2—Suppression of Tumorigenicity 2; WBC—White blood cell.

## Data Availability

The data presented in this study are available on request from the corresponding author.
